# Analysis of Area-Specific Expression Patterns of RORbeta, ER81 and Nurr1 mRNAs in Rat Neocortex by Double In Situ Hybridization and Cortical Box Method

**DOI:** 10.1371/journal.pone.0003266

**Published:** 2008-09-25

**Authors:** Junya Hirokawa, Akiya Watakabe, Sonoko Ohsawa, Tetsuo Yamamori

**Affiliations:** 1 Division of Brain Biology, National Institute for Basic Biology, Okazaki, Japan; 2 Department of Basic Biology, The Graduate University for Advanced Studies, Okazaki, Japan; Indiana University, United States of America

## Abstract

**Background:**

The mammalian neocortex is subdivided into many areas, each of which exhibits distinctive lamina architecture. To investigate such area differences in detail, we chose three genes for comparative analyses, namely, RORbeta, ER81 and Nurr1, mRNAs of which have been reported to be mainly expressed in layers 4, 5 and 6, respectively. To analyze their qualitative and quantitative coexpression profiles in the rat neocortex, we used double in situ hybridization (ISH) histochemistry and cortical box method which we previously developed to integrate the data of different staining and individuals in a standard three-dimensional space.

**Principal Findings:**

Our new approach resulted in three main observations. First, the three genes showed unique area distribution patterns that are mostly complementary to one another. The patterns revealed by cortical box method matched well with the cytoarchitectonic areas defined by Nissl staining. Second, at single cell level, RORbeta and ER81 mRNAs were coexpressed in a subpopulation of layer 5 neurons, whereas Nurr1 and ER81 mRNAs were not colocalized. Third, principal component analysis showed that the order of hierarchical processing in the cortex correlates well with the expression profiles of these three genes. Based on this analysis, the dysgranular zone (DZ) in the somatosensory area was considered to exhibit a profile of a higher order area, which is consistent with previous proposal.

**Conclusions/Significance:**

The tight relationship between the expression of the three layer specific genes and functional areas were revealed, demonstrating the usefulness of cortical box method in the study on the cerebral cortex. In particular, it allowed us to perform statistical evaluation and pattern matching, which would become important in interpreting the ever-increasing data of gene expression in the cortex.

## Introduction

The mammalian neocortex consists of many areas that are defined on the basis of unique connectional and functional properties [1], [2]. In accordance with functional specialization, these areas exhibit various differences in terms of their structural configurations as revealed by Nissl staining and other conventional histological techniques [1], [3]. More recently, it has become possible to selectively visualize particular neocortical structures by techniques to map gene products, such as immunocytochemistry [4], [5], receptor autoradiography [6]–[9], and in situ hybridization histochemistry (ISH) [10]–[12]. For example, several genes have been shown to exhibit layer- and area-specific expression profiles during development or in adulthood [5], [10], [11], [13]–[23]. For the rodent cortex, it is now possible to examine the expression data of most of the known genes in public databases [24]–[27]. Effective use of such information may enable us to reveal apparently hidden structures of neocortical areas, such as new sublayers and areas defined by expression of a unique set of genes.

In our previous study, we have shown that layer-specific gene expressions can reveal cortical structures across areas and species [23]. Consistent with the six-layer model originally proposed by Brodmann [1], the lamina expressions of several genes were conserved across areas in monkey and mouse neocortices. At the same time, we observed various area differences in their expression patterns. For example, we observed that the width and intensity of gene expressions exhibit abrupt changes across the V1–V2 border in the monkey cortex. We also found that a subtype of excitatory neurons that express 5-HT2C receptor mRNA are localized in layer 5 in most areas, but in layer 6 in monkey V1. These observations may reflect the conspicuous difference of monkey V1 compared with other areas [28]–[30]. While the differences between V1 and V2 are rather conspicuous, there are often more subtle differences in other areas. These subtle differences are more difficult to analyze, owing to many factors, including staining artifacts and sample-to-sample variabilities.

The simultaneous visualization of two different staining patterns may circumvent this problem to some extent by providing a reference to analyze the other. Accumulating samples for quantitative evaluation may also be helpful. Nevertheless, the latter method requires that data are obtained and accumulated from accurately identified cortical areas. This task is, in fact, quite difficult, especially for the rodent cortex, where there are no clear-cut borders for area demarcation. In an effort to analyze the spatial distribution of *c-Fos* expression [31], we previously developed cortical box method. By this method, the gene expression in the rat neocortex can be mapped into a three-dimensional standardized cortical box from serially prepared sections. Importantly, this standardization process enables us to integrate data from different animals for statistical evaluation. Several methods have been proposed to reconstruct section data into a three-dimensional structure (e.g., [27], [32]–[35]). In comparison with these previous methods, the advantage of our method is that the lamina information is preserved in one axis of the three-dimensional cortical map. The simplicity of the result is also a strength of our method, which helps in the intuitive understanding of the area distribution.

In the present study, we used the rat cortex as a model system to analyze the area-specific expression patterns of three “layer-specific” mRNAs, RORbeta [14], ER81 [10], [22], [23] and Nurr1 [13], [19], [23], which are expressed mainly in layers 4, 5 and 6 of the rodent neocortex, respectively. Although the heterogeneous expressions of these genes within neocortical areas have been reported in these previous studies, we think that more detailed analysis is necessary to understand their complex spatial distribution patterns. In this endeavor, we employed double ISH to examine the coexpression profiles of these genes at single cell level and cortical box method for a global view. Cortical box method enabled us to perform statistical evaluation of the data from different individual animals as well as multivariate analysis to extract common and differential patterns of expression for the three genes. Our study underscores the usefulness of quantitative approaches in analyzing gene expression data.

## Results

### Heterogeneity of layer-specific gene expression revealed by double ISH

**Figure 1:**
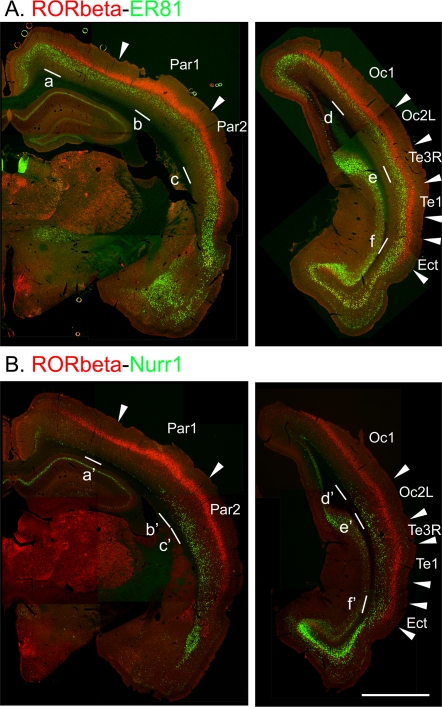
Double in situ hybridization histochemistry (ISH) of RORbeta/ER81 (A) and RORbeta/Nurr1 (B). Signals in red are for RORbeta and those in green are for ER81 (A) or Nurr1 (B). The arrowheads indicate the area borders that were deduced by comparing the gene expression patterns shown by double ISH and those revealed by the cortical box method. Par1, Par2, Oc1, Oc2L and Te1 correspond to the primary and secondary somatosensory areas (Par1 and Par2), the primary and secondary visual areas (Oc1 and Oc2L) and the primary auditory area (Te1). Ectorhinal cortex (Ect) is also indicated. The white bars denoted as a–e and a'–e' are the regions magnified in Fig. 2. This figure is a montage of several images. Although the lighting condition of the original images was not even at this low resolution, we adjusted the contrast of each component image manually so that the montage appeared to be consecutive. Scale bar, 2 mm.

**Figure 2:**
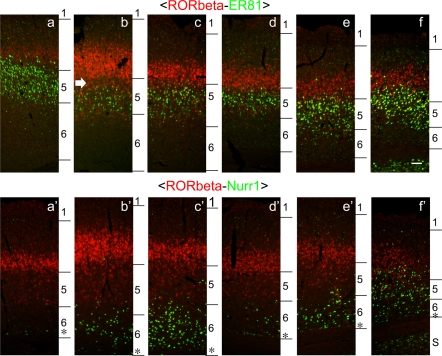
Area differences in gene expressions. The regions denoted in Fig. 1 are magnified. In these figures, the contrast was adjusted simultaneously so that the area differences can be directly compared across different areas. The layers denoted on the left side of each panel were determined in reference to the Hoechst 30442 nuclear staining. These panels are considered to correspond to cytoarchitectonic areas as follows: a; Oc2MM, b; Par1, c; Par2, d; Oc1, e; Te3R, f; Ect, a'; DZ, b'; Par1/Par2 border, c'; Par2, d'; Oc1, e'; Oc2L, f'; Oct. S; subiculum. Scale bar: 100 µm.

Figure 1A shows the double ISH of RORbeta and ER81 mRNAs and Fig. 1B shows that of RORbeta and Nurr1 mRNAs in the middle and occipital coronal sections of rat brains. RORbeta and Nurr1 mRNAs showed prominent area differences while ER81 mRNA did not show such conspicuous area difference. As reported previously [14], RORbeta mRNA was most abundant in the barrel field of the parietal cortex area 1 (Par1) (Fig. 1). RORbeta mRNA was generally expressed more abundantly in the sensory areas than in other areas. ER81 mRNA exhibited the opposite pattern, showing higher levels of expression in the areas where the RORbeta mRNA expression level was low (Fig. 1A, see also Fig. 2, panels a, e and f). Nurr1 mRNA exhibited a characteristic area expression pattern (Fig. 1B): its expression in layer 6A was restricted to the lateral regions (e.g., Fig. 1B, par2, Oc2L and Te1, see also Fig. 2, panels b', c', e' and f') and there was only low expression in layer 6B in the dorsal areas (e.g., Fig. 1B, Par1 and Oc1, see also Fig. 2 panels a' and d').

Figure 2 shows the double ISH of these genes in various areas at higher magnification. At this magnification, we were able to identify individual neurons and examine how the positively stained neurons are distributed within and across layers in different areas. For example, although Nurr1-mRNA-positive cells were mostly confined to layers 6A and 6B of most areas (Fig. 2, panels a'–e'), its subpopulation was found scattered into layer 5 and even layer 4 in the lateral-most areas. In the laterocaudal area (e.g., Fig. 1B, Ect), Nurr1-mRNA-positive cells were found both in layers 5 and 6 to a similar extent and extensively intermingled with the RORbeta-mRNA-positive cells (Fig. 2, panel f'). Such area differences were also observed for ER81-mRNA-positive cells. In the barrel field (Fig. 2, panel b), we observed a sublayer with lower expression levels of both RORbeta and ER81 mRNAs (white arrows). Based on Hoechst nuclear staining, this cleft sublayer appears to be the upper part of layer 5 (data not shown), despite low number of ER81-mRNA-positive cells. Such a gap does not exist in other areas (Fig. 2, panels e and f). In the laterocaudal area (e.g., Fig. 1B, Ect), neurons that expressed both ER81 and RORbeta mRNAs at moderately high level were located around the border between layers 4 and 5 (Fig. 2, panel f). These observations indicate heterogeneous lamina expression patterns of the “layer-specific” genes at the cellular level.

**Figure 3:**
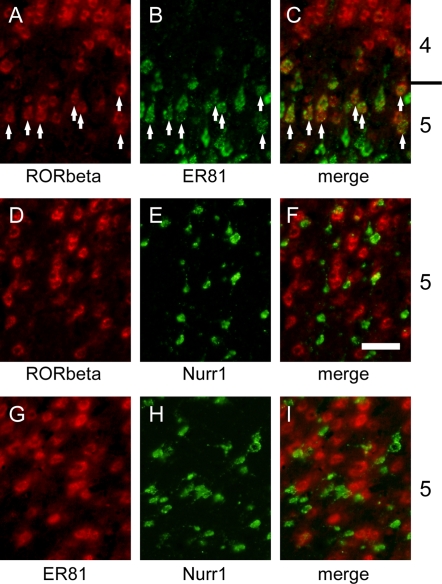
Coexpression of RORbeta, ER81 and Nurr1 genes. (A)–(C) Double ISH of RORbeta (red) and ER81 (green) mRNAs in a somatosensory area. Note the extensive coexpression of the two genes (denoted by the arrows). (D)–(F) Double ISH of RORbeta (red) and Nurr1 (green) mRNAs in a laterocaudal area. (G)–(I) Double ISH of ER81 (red) and Nurr1 (green) mRNAs in a laterocaudal area. Note that the two genes in (D)–(F) and (G)–(I) are not coexpressed. Scale bar: 50 µm.

The overlapping mRNA expression profiles raised the possibility that the two mRNAs with different lamina specificities are coexpressed in the same cells. This was examined in high-magnification photos in Fig. 3. Although RORbeta is a marker for layer 4, it is also expressed in layer 5 throughout the rat neocortex. When we examined the coexpression of RORbeta and ER81 mRNAs by double ISH, these mRNAs were coexpressed within the same cells in layer 5 (Fig. 3A–3C). Layer 4 neurons generally expressed only RORbeta mRNA, while many layer 5 neurons expressed only ER81 mRNA. However, the layer 5 neurons expressing RORbeta mRNA mostly coexpressed ER81 mRNA. Such coexpression was observed in all the areas examined (data not shown). On the other hand, RORbeta and Nurr1 mRNAs were not coexpressed in the same neurons even in the areas with extensive intermingling (Fig. 3D–3F). Similarly, ER81 and Nurr1 mRNAs were not expressed within the same neurons (Fig. 3G–3I). This point has been shown previously for mice [23], but our data now showed the same co-expression pattern in rats. Thus, we conclude that the coexpression preferences of the three genes are common across areas, although the relative abundance and distribution are quite divergent.

### Cortical box method for quantitative analysis of area-specific gene expression

**Figure 4:**
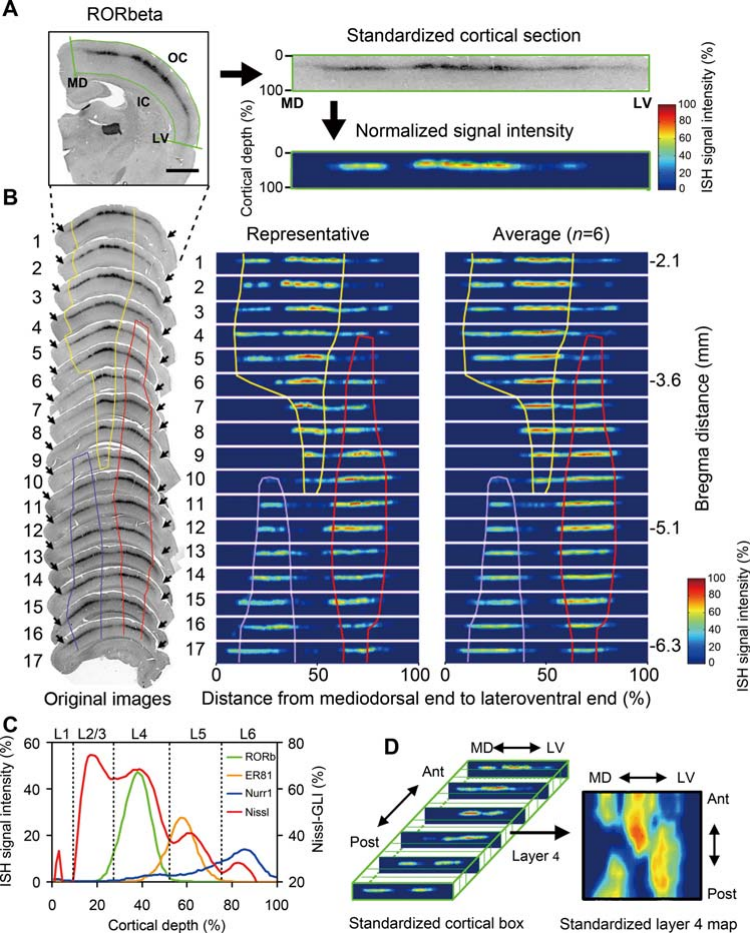
Cortical box method reveals the area-specific expression of RORbeta mRNAs. (A) Example of a cortical section of RORbeta ISH image processed for the cortical box standardization procedure. The mediodorsal end (MD), lateroventral end (LV), inner contour (IC), and outer contour (OC) were manually determined to select the part of the cortex for further processing. The selected cortical region was converted into a standard rectangle (a standardized cortical section). The intensity of the ISH signals was normalized and pseudocolored, so that the mean +1 SD becomes 0% and the mean +3 SD becomes 100% (see Materials and Methods for details). Scale bar, 2 mm. (B) Example of one series of coronal sections (from the Bregma distance of −2.1 to −6.2 mm, number 1 to 17) of RORbeta ISH (left). These images of cortical sections were standardized as displayed on the right side of the original images (representative). We performed the same processing for six series of such samples (right and left hemispheres from three rats) and averaged them. Note that the patterns of the representative and the average data are quite similar. We also performed the same procedure for the Nissl-stained samples and determined the cytoarchitectonic borders for primary somatosensory (Par1, yellow), visual (OC1, blue) and auditory (Te1, red) areas. (C) Layer distributions of RORbeta, ER81 and Nurr1 (left axis) as well as the Nissl-gray level index (GLI, right axis) from the pial surface (0% of cortical depth) to the cortex/white matter border (100%). Each line plot shows the average signal intensity at a given cortical depth. The entire cortical regions except the mediodorsal and laterocaudal 10% were used to calculate the average. Green, orange, blue and red lines represent RORbeta, ER81, Nurr1 ISHs and Nissl staining, respectively. (D) Conceptual figure to illustrate the construction of the cortical box. In this example, the layer 4 fraction (30–50% cortical depth) was extracted to demonstrate the area distribution pattern of RORbeta mRNA in a two-dimensional map.

As mentioned in the introduction, it is difficult to accurately identify cortical areas without clear-cut landmarks. To circumvent this problem, we applied a standardization and reconstruction procedure for the ISH samples of the serially prepared coronal sections of the posterior part of the rat cortex as follows (see also [31]). In the reconstruction, the shape of the cortex was transformed to fit into a rectangle, as illustrated in Fig. 4A. The left and right borders of the rectangle correspond to the medial ends of the cortex and the rhinal fissure, respectively, both of which can be easily determined. We also normalized the level of ISH signals so that the relative strength of the ISH signals at a given location can be compared across different data sets (Fig. 4A). Figure 4B illustrates the standardization process from the ISH data of the RORbeta gene. As shown in this figure, seventeen ISH coronal sections in total were used to cover the posterior part of one rat brain hemisphere (−2.1 to −6.3 mm from Bregma) with 280 µm intervals (Fig. 4B; original images). It was already evident from the original images that there are three distinct clusters of high RORbeta signals, which roughly corresponded to the somatosensory, auditory and visual areas (delineated by yellow, red and blue lines, respectively). The middle panel of Fig. 4B shows the images transformed into seventeen rows of cortical rectangles. In these rows of images, the three clusters of high RORbeta ISH signals were now more clearly visualized (“Representative”). Importantly, once the staining intensity was standardized, we could easily integrate multiple sets of data. In the right panel of Fig. 4B, the average of six sets of samples from three rats is shown. Note that the pattern of a single set of sample (“Representative”) was very similar to that of the average. The characteristic expression pattern of RORbeta mRNA is therefore reproducibly captured across different animals.

In the final step of image processing, we arrayed the seventeen cortical rectangles, so that the posterior cortex is three-dimensionally reproduced as a box (Fig. 4D). By this procedure, the expression data are now mapped onto a standardized cortical box, which can be easily manipulated for various analyses. One application of the cortical box method is to show gene expression at a given lamina position as a two-dimensional “layer map”, which represents a map of a virtual tangential section (Fig. 4D). To make maps for layers 2/3, 4, 5 and 6, we first determined the borders of these layers based on Nissl staining, as well as the ISH patterns of the three layer-specific genes, RORbeta, ER81 and Nurr1. Figure 4C shows the lamina distribution of the Nissl-grey level index (GLI) [36]–[38] and that of the three mRNAs, averaged over the central portion of the standardized cortical box (see Materials and Methods). We observed four distinct peaks in the Nissl-GLI, which are considered to correspond to the cytoarchitectonic layers 2/3, 4, 5 and 6. As expected, the latter three peaks coincided very well with the peaks of RORbeta (around cortical depth of 30–50%), ER81 (around cortical depth of 50–75%) and Nurr1 (around cortical depth of 75–100%) (Fig. 4C). Within the regions defined in our method, we observed little variance in the positions of the lamina borders. On the basis of this data, we determined the lamina borders to make layer maps that are described in the following sections.

**Figure 5:**
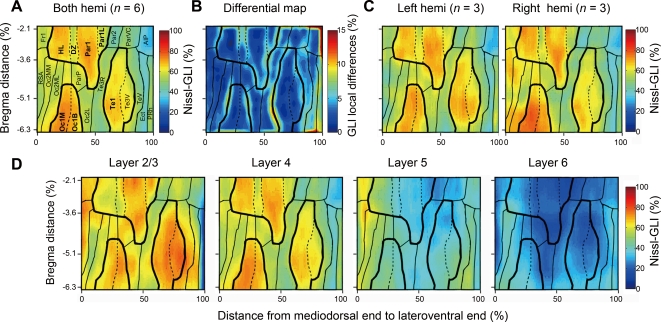
Spatial distribution of Nissl-GLI in standardized cortical maps. (A) Averaged standardized layer 4 map of the Nissl-gray level index (GLI) was constructed from both hemispheres of three animals. (B) The areal borders were determined on the basis of the differential map, which shows the local differences of the GLI values. (C) Standardized layer 4 maps of GLI constructed using the data from the left and right hemispheres of three animals. The two independent maps exhibit very similar patterns despite no overlaps in the samples used for image processing. (D) Nissl GLI maps for different layers. AIP, agranular insular cortex, posterior part; DZ, parietal cortex, area 1, dysgranular zone; Ect, ectorhinal cortex; Fr1, frontal cortex, area 1; HL, parietal cortex, hindlimb area; Oc1B, occipital cortex, area1, binocular part; Oc1M, occipital cortex, area 1, monocular part; Oc2L, occipital cortex area 2, lateral part; Oc2ML, occipital cortex, area 2 mediolateral part; Oc2MM, occipital cortex, area 2, mediomedial part; Par1, parietal cortex, area 1; Par1L, parietal cortex, area1, lateral part; Par2, parietal cortex, area2; ParP, parietal cortex, posterior area; ParVC, parietal cortex, ventral area, caudal part; PRh, perirhinal area; RSA, agranular retrosplenial cortex; Te1, temporal cortex, area 1; Te3R, temporal cortex, area 3, rostral part; Te3V, temporal cortex, area 3, ventral part; TeV, temporal cortex ventral area.

To relate the expression patterns of the three layer-specific genes to the cytoarchitectonic areas determined by the standard method, we applied the cortical box method to Nissl staining using the GLI, which has been previously used to define area borders [36]–[38]. Figure 5A shows the layer 4 maps of Nissl-GLI obtained from three different rats. In these maps, three clusters of high Nissl-GLI were observed, which were considered to correspond to somatosensory, auditory, and visual areas (Fig. 5A; bordered by thick lines). The GLI distributions were not homogeneous within the three clusters and we could draw potential borders for the subareas (solid and dashed lines). These borders were semi-automatically determined on the basis of the differential map (Fig. 5B, see Materials and Methods). The locations of the area borders determined in this way generally well matched those of the standard atlases [8], [39] (Fig. 5A) (see Discussion for detailed comparisons with standard atlases).

The borders in Fig. 5A are determined from the average of six different sets of Nissl-stained samples. The layer maps of the right and left hemispheres (n = 3 each) are shown separately in Fig. 5C. Although there are some variances between these two maps (e.g., compare the mediodorsal regions of the middle and right panels of Fig. 5C), we were able to determine the borders that match well for both sets of samples by averaging the data. The maps of other layers also suggest the consistency of the area borders determined from the layer 4 map (Fig. 5D). For example, the expression changes in the layer 2/3 map generally occurred at the same border as that in the layer 4 map. However, there were several important differences in the patterns. For example, the GLIs of the mediodorsal areas (agranular retrosplenial cortex (RSA) and frontal cortex, area 1 (Fr1)) were high in layer 2/3 but not in layer 4. Also, the GLI in area HL (hind limb), a subregion of the somatosensory areas, was low in layers 2/3 but high in layer 4. Despite such differences in the area distribution patterns between layers, the borders for the changes in the GLI were the same for layers 2/3 and 4. Similarly, the GLIs of layers 4 and 5 were mostly complementary and the same borders were observed. Thus, the Nissl-GLI is considered to faithfully reflect the cytoarchitectonic area map.

### Analysis for area-specific distribution patterns of RORbeta, ER81 and Nurr1 mRNAs by cortical box method

**Figure 6:**
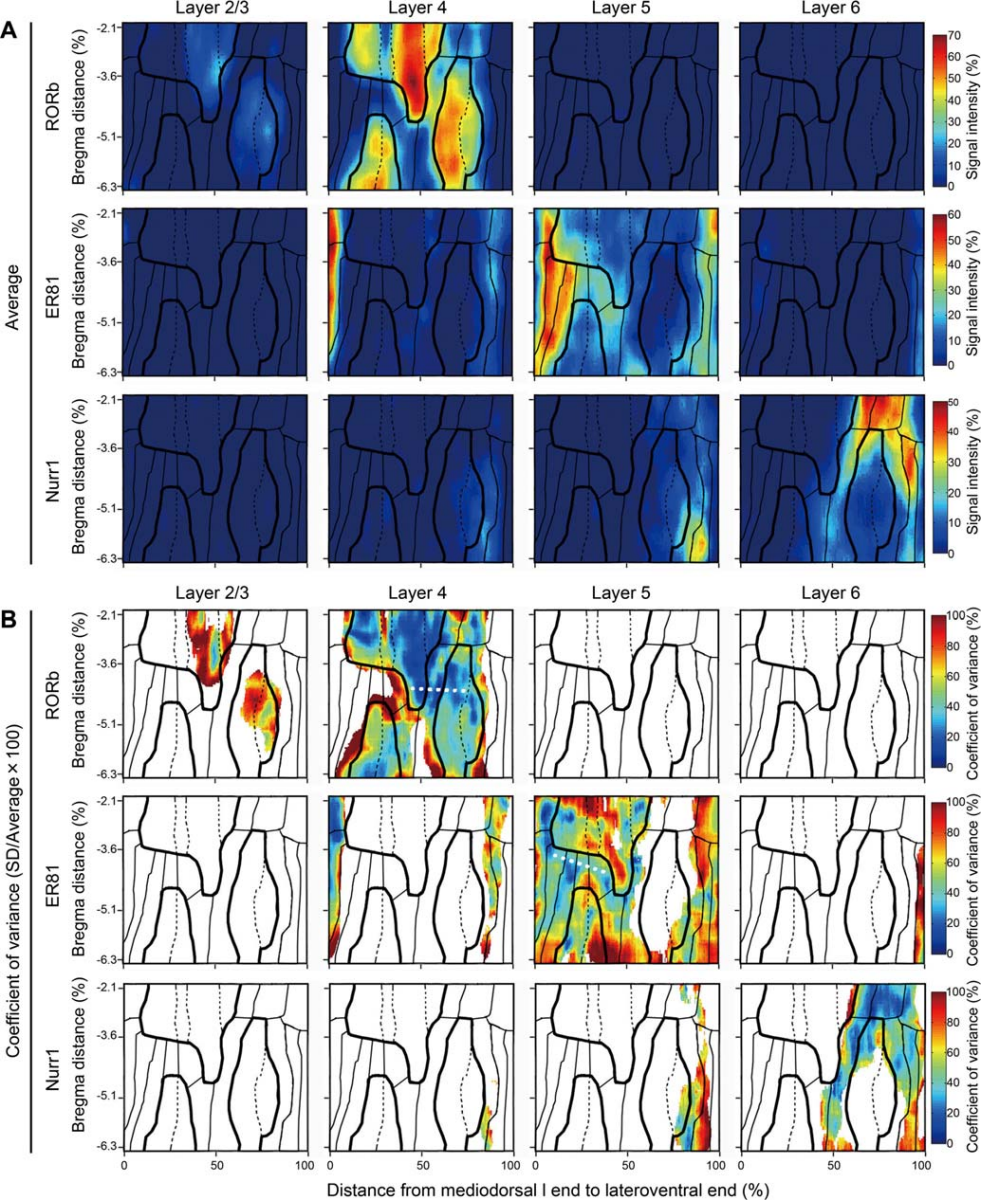
Spatial distributions of RORbeta, ER81 and Nurr1 in the standardized cortical map. (A) Average standardized layer maps (layers 2/3, 4, 5 and 6) for in situ hybridization immunohistochemistry (ISH) of RORbeta, ER81 and Nurr1. Black lines indicate the cytoarchitectonic borders of the cortical area defined in Fig. 5A. (B) Coefficient of variance (CV) of standardized layer maps (layers 2/3, 4, 5 and 6) for in situ hybridization immunohistochemistry (ISH) of RORbeta, ER81 and Nurr1. White color represents the CV values of the pixels with low average values (see Materials and Methods).

Following the determination of the cytoarchitectonic lamina and area borders by Nissl staining, we analyzed the distribution patterns of RORbeta, ER81 and Nurr1 mRNAs by the cortical box method. Figure 6A shows the standardized maps of RORbeta, ER81 and Nurr1 mRNAs for layers 2/3, 4, 5 and 6. The ISH signals of these mRNAs were mostly observed in layers 4, 5 and 6, respectively, as expected. In addition, we clearly observed area-specific distribution patterns for all the three mRNAs. These patterns coincided well with the borders determined by Nissl staining (Fig. 6A; solid and dashed lines). Actually, the spatial distribution of RORbeta mRNA was very similar to that of Nissl-GLI in layer 4 (r = 0.63, P<0.01, linear regression model analysis), and the spatial distribution of ER81 was similar to that of Nissl-GLI in layer 5 (r = 0.70, P<0.01, linear regression model analysis). However, the area difference between RORbeta and ER81 mRNAs was much larger than that expected from the Nissl-GLI. Besides, the area distribution of Nurr1 mRNA was different from that of the Nissl-GLI in layer 6, although they seemed to show the same borders.

The layer maps also showed area-specific differences in the lamina specificity of these genes. For example, the expression level of RORbeta mRNA was higher in layers 2/3 of the Par1 subfield of the somatosensory cortex and the auditory cortex, which is consistent with the findings described in a previous report [8]. Furthermore, the expression levels of Nurr1 in the temporal cortex ventral area (TeV) and ectorhinal cortex (Ect) (about 90% of the mediodorsal distance at the bregma distance of −6 mm) were higher in layer 5 rather than layer 6, which is consistent with results of the double ISH (Fig. 2, panel f'). Although ER81 mRNA exhibited a shift of expression from layer 5 to layer 4 in the medial-most retrosplenial area (RSA), this may be due to the difference in the overall lamina position in this area.

An advantage of our method is that the variability of gene expression across different sets of samples can be quantitatively estimated. In Fig. 6B, we mapped the coefficient of variance (CV) of each gene for each layer map. CV is the percentage of the standard deviation (SD) per average and a measure of the relative variability. By definition, CV becomes unreliable when the average is small. Thus, we excluded the areas with low gene expression values from the analyses in Fig. 6B (white areas). The map shows that the CVs were generally low (<50%) in the central regions of a cluster with high average values and were high (>80%) in the borders of those clusters. This result suggests that there is little sample-to-sample variance in the area distribution of these genes, and that the variability is concentrated at the borders. The CV showed a constantly low level in the transition regions from area Par1 to the temporal cortex, area1 (Te1), in the layer 4 map of RORbeta and from area Oc2MM to Oc2L in the layer 5 map of ER81 (Fig. 6B; white dotted lines), despite large changes in the averages. In these regions, even the area borders are reproduced across different animals. This demonstrates the high reliability of the gene expression mapping in our method.

### Common and different characteristics of various cortical areas captured by multivariate analyses

**Figure 7:**
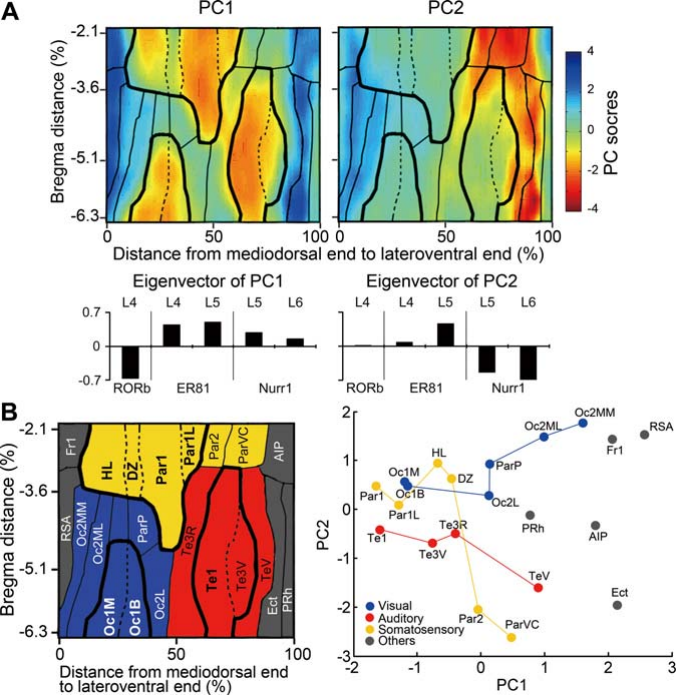
Principal component analysis (PCA) of standardized cortical map. Cortical areas were divided into 340×100 points so that the ISH signal values could be represented by a matrix of 34000 data points. The data of five layer maps (layer 4 of RORbeta, layers 4 and 5 of ER81, and layers 5 and 6 of Nurr1) were analyzed for PCA to extract two primary components, PC1 and PC2. (A) Pseudocolored layer maps (top) and eigenvectors (bottom) of PC1 and PC2. The layer maps indicate the PC scores plotted in the two dimensional space. The eigenvectors below the layer maps show the contributions of the five layer maps in constructing each PC. (B) The averaged PC1 and PC2 scores of each cortical area (shown in the left panel) were plotted in the PC1–PC2 space. The cortical areas represented by the dots in the right panel are grouped by modality and connected by colored lines (yellow, red and blue for somatosensory, auditory and visual, respectively) in the order of PC1 scores. Gray represents other areas, including motor, limbic and paralimbic areas. The nomenclature of each area is the same as that in Fig. 5.

The distribution pattern shown in Fig. 6A suggests that the RORbeta mRNA expression level is high in the sensory areas, whereas the ER81 and Nurr1 mRNA expression levels are generally high in the areas with low RORbeta mRNA expression level. This observation suggests that the distribution patterns of these mRNAs may be governed by some common rules. In an attempt to discover such rules, we performed principal component analysis (PCA) using five layer maps of RORbeta (layer 4), ER81 (layers 4 and 5) and Nurr1 (layers 5 and 6). Other layer maps were excluded from the analysis, because there are very little signals, if any, in other maps, and not reliable. In this analysis, we first divided each map into 1000×340 blocks, so that the spatial maps as shown in Fig. 6A could be represented by rows of data having 34,000 data points. When five of such datasets are combined, it is considered as 34,000 points plotted in a five-dimensional space. The purpose of the PCA is to find new “axes” to explain the variability of the 34,000 data points with the least variables. In other words, we expected PCA to decompose the five maps of spatial distribution data into fewer maps that represent the common features of spatial variations. Figure 7A shows the first two principal components (PCs) obtained by PCA. The eigenvector of each PC is graphed at the bottom. These bar graphs demonstrate the contribution of each layer map in determining the PC scores that are illustrated as the colored maps on top. At first glance, PC1 is similar to the layer 4 map of RORbeta, while PC2 is similar to the layer 6 map of Nurr1. However, as the bar graphs indicate, PC1 and PC2 have contributions from all the five layer maps in various degrees and directions. For example, in addition to RORbeta patterns, the ER81 patterns in layers 4 and 5 considerably contributed to PC1, but they were in the reverse direction. This means that PC1 represents a feature shared by RORbeta and ER81 (and Nurr1 to a lesser extent), which is shown as their complementary distribution patterns. Similarly, PC2 represents a feature shared by layer 5 of ER81 and Nurr1, but in a complementary manner. There are almost no contributions of RORbeta in this component.

In an attempt to decipher the meaning of the two axes represented by PC1 and PC2, we calculated the average scores of these PCs in cytoarchitectonically determined cortical areas and plotted them in the PC1–PC2 space (Fig. 7B). In this figure, the cortical areas were roughly classified into four categories. Somatosensory, auditory, and visual domains were colored in yellow, red and blue, respectively. The medial and lateral ends of the cortex, which are colored in grey, are motor, limbic and paralimbic areas and considered to be higher areas in the cortical hierarchy in terms of sensory inputs. In the plot, the areas with the same modalities are grouped by lines to aid in the visualization. Here, we observed two features. First, the primary sensory areas (Par1, Te1, Oc1) were clustered at the lowest value of PC1 with little contribution from PC2. The higher order multisensory areas tended to be located in the higher values of PC1. Second, the higher order association areas of different modalities were dispersed in the plot, because of the contribution of different PC2 values. Together, these observations suggest the similarity of the primary sensory areas and the diversion of association areas, as far as the expression of the three “layer-specific” genes are concerned.

## Discussion

We studied the area-specific expression patterns of three layer-specific genes, RORbeta, ER81 and Nurr1, using double ISH and the cortical box method [31]. Double ISH showed that the coexpression profiles of these genes are the same across areas, whereas their relative abundance and the extent of intermingling differ considerably. The cortical box method allowed us to quantitatively and objectively analyze the three-dimensional pattern of gene expression using integrated ISH data sets. We first discuss the methodological aspects of our study and then the implications of our findings in terms of the area architecture of the rat cortex.

### Cortical box method is a useful tool for gene expression analyses

For histological studies, it is critically important to accurately identify various anatomical structures. This is particularly true for the study of the neocortex that consists of many areas and subareas. Traditionally, the Nissl staining patterns have been used as criteria for discrimination of cytoarchitectonic areas (e.g., [1],[3]). Nevertheless, the area differences determined by Nissl staining are often subtle and susceptible to various artifacts, such as inhomogeneous staining and sampling variances. Although quantitative methods of characterizing cortical areas [36]–[38], [40] enable observer-independent area demarcation, the area identification is still subtle and requires exact spatial information of the section of interest. In cats and monkeys, sulcal landmarks help in area identification. However, the rodent cortex offers no such landmarks. To circumvent this problem, we previously developed the cortical box method to analyze c-fos immunoreactivity [31], which we now applied to the analysis of ISH data. This method uses a set of coronal sections that cover the entire posterior cortex of the rat. With sufficient numbers of sections, it is possible to accurately estimate the continuity of the areas that span several sections (see Fig. 3A). Because this method transforms expression data into a standardized form, many different sets of data can be compared quantitatively. Furthermore, although the selection of ROI (region of interest) for standardization is still determined manually, this process only requires the determination of the medial and lateral ends of the cortex, which have clear landmarks and are unambiguous. The effectiveness of these features is demonstrated by the reproducibility of cytoarchitectonic areas (Fig. 5A) and low CV of the gene expression data (Fig. 6B) across different sets of samples. As we have shown in this study, this method is applicable to the sections stained by various methods including Nissl staining, immunohistochemistry, ISH and possibly other methods, such as neural tracer dyes. This method will, thus, enable us to integrate different types of histological data in the same coordinate for quantitative analyses.

In the current study, we pooled the data from both right and left hemispheres of three different rats, to reduce experimental variability. Although there is a possibility that the two hemispheres show differences in gene expression, we were not able to find sign of lateralization, under the current number of dataset (n = 3). However, a study using larger number of datasets for cortical box analysis may clarify if any lateralization exists in rodent neocortex.

Many methods have been developed to standardize the rodent brain map (e.g., [27], [32], [33], [35], [41], [42]). In particular, Gabbott et al. (2005) reported a method similar to the cortical box method to investigate the cortical projections from the rat frontal areas [35]. Our method is also conceptually similar to the surface-based atlases developed by Van Essen and coworkers [43]. The advantage of these methods is that by flattening the three-dimensional cortex, it becomes easy to understand intuitively the global picture of gene expression. It is also important that the cortical box method enables the quantitative analyses of the spatial distribution data. Even when there are sample-to-sample variances owing to various reasons, we can extract useful information by averaging the data. We can also estimate the significance of such variances (see Fig. 6B). We noted that the variability of gene expression were concentrated at area borders (Fig. 6B), which could be attributable to individual difference of the area architecture, although it is possible that such variance was derived from experimental variance. One advantage of our method over other flattening techniques is the simplicity and ease of use, because it is optimized for the simple sulcus structure of the rodent posterior cortex. On the other hand, it would be difficult to directly apply it to the convoluted cortex of other mammalian species. For example, because the thickness of each lamina varies greatly in areas for primate brains (e.g., see Fig. S3 of [23]), it will become difficult to construct layer maps using the same layer borders for different areas. However, if we are to limit the analysis to a subregion of the cortex that can be defined by clear-cut landmarks (such as sulci), cortical box method may be used to analyze convoluted brains as well.

Despite the many useful features of the cortical box method as pointed above, caution is required when interpreting the data, because it does not offer information on the expression at the cellular resolution level. For example, in the layer 6 maps of ER81 and Nurr1, the maps of two genes overlapped in the laterocaudal areas (Figs. 5 and 6, TeV and Ect), although the double ISH results demonstrated that they were not coexpressed (Fig. 3G–I). It is also notable that, owing to normalization, the low expression levels tend to be ignored in a global view. For example, although RORbeta mRNA is clearly expressed by layer 5 neurons throughout areas (Fig. 2), it does not show up in the layer 5 map (Fig. 6A). In the case of ER81, the very high expression level in the medial areas obscures its widespread distribution across the entire neocortical areas in layer 5. The cortical box method needs to be coupled with careful analyses of expressions at the cellular level. It should also be noted that, by fitting the cortex into rectangle, the information of the cortical thickness is lost, although the relative expression value is preserved throughout layers. To examine the differential cortical thickness across areas, other analytical method needs to be used.

### Identification of cortical areas by cortical box method

In previous studies, cortical areas were successfully delineated in an objective manner by Nissl-GLI analyses [8], [36]–[38]. Therefore, we relied on Nissl-GLI patterns to identify cytoarchitectonic areas in our standard three-dimensional space. The layer 4 map of Nissl-GLI was consistent overall with the Nissl-GLI map of Zilles and coworkers [8]. However, there were several points wherein we incorporated the area classification by other researchers. For example, we found a subarea “Par1L” in the lateral region of the primary somatosensory cortex, which is not shown in the original map of Palomero-Gallagher and Zilles [8]. Judged from the location, Par1L seems to correspond to the representation region of the upper lip, which was recently noted by others [39]. We also found another subarea “dysgranular zone (DZ)” between areas HL and Par1. This subarea appears to correspond to the most medial zone of a matrix of dysgranular cortex into which the barrels are embedded [44]–[47]. The DZ appears to partially overlap with “FL” in the map of Palomero-Gallagher and Zilles [8]. Although the borders of DZ were not determined by GLI analysis [8], our data showed that they extended anteroposteriorly along HL. The consistency with the RORbeta gene expression supports this area delineation. In the auditory cortex, we distinguished Te1 and Te3V, on the basis of Nissl-GLI and RORbeta expression. Te3V in our map is considered to correspond to the belt region of the auditory cortex [8], [48]. Ventral to TeV, we delineated Ect according to Paxinos and Watson [49] and Swanson [50]. We emphasize that the cytoarchitectonic areas determined by Nissl-GLI are well consistent with the expression patterns of the layer-specific genes, validating the area demarcation by our method.

### Significance of area-specific gene expression

Previous studies using receptor autoradiography have revealed that the brain's chemoarchitectonic organization is correlated with cyto- and myeloarchitectonical organizations [7], [51]. Our analysis also revealed tight correlation between gene expression and the cytoarchitectonic area. In particular, RORbeta and ER81 patterns were quite similar to those of Nissl-GLI in layers 4 and 5, respectively. This is anticipated to some extent, because both Nissl-GLI and gene expression intensity should positively correlate with the neuronal density. However, RORbeta and ER81 mRNAs were expressed by a subpopulation of cortical neurons and the variations in their densities appeared to be much greater than those expected from the neuronal density of the layer of interest (Fig. 2). Besides, the perceived intensity of labeling per cell also varied across areas. Regarding Nurr1 gene expression, the positive cells represent only a minor population of the layer 6 neurons, and the area distribution pattern was completely different from that of the Nissl-GLI. These observations suggest that there may exist some rules that commonly affect their area-specific expression patterns other than neuronal density. By using PCA, we tried to find such rules and obtained two principal components (PC1 and PC2).

The PC1–PC2 plot of different cortical areas shown in Fig. 7B indicates the features of these areas characterized by RORbeta, ER81 and Nurr1 gene expressions. This figure clearly shows that the primary sensory areas, Par1, Par1L, Te1, Oc1M and Oc1B, cluster together at lower PC1 values, while the higher sensory areas and multimodal areas are away from the “cluster” of primary sensory areas with higher PC1 values, but with various PC2 values. This pattern demonstrates the similarity of gene expression in the primary sensory areas, as well as the diversity of gene expression patterns in the higher sensory areas. There are several possible explanations for the similarity of the primary sensory areas. First, the primary sensory areas generally have “granular” layer 4, which contains a high density of thalamorecipient neurons. The PC1 score may be positively or negatively correlated with the neuronal density as we discussed earlier. Second, the primary sensory areas receive strong inputs from the primary sensory thalamus [52], [53], which can be visualized by cytochrome oxidase staining [54], [55]. It is well known that certain genes exhibit activity-dependent regulation during development or in the adult [17], [56]–[58]. The expressions of RORbeta, ER81 and/or Nurr1 genes may be affected, directly or indirectly, by the thalamocortical inputs and contribute to the low PC1 scores in the primary sensory areas. In this context, it is interesting that area DZ exhibits a high PC1 score despite its location within Par1. Previous studies suggest that the dysgranular zones in barrel cortex including our DZ defined here does not receive inputs from the primary somatosensory thalamic nucleus; instead it receives inputs from a higher order thalamic nucleus and adjacent primary somatosensory areas as well [45]–[47], [59], [60]. The high PC1 score is consistent with this observation and may support a proposal that the dysgranular non-barrel cortex is a higher order somatosensory area [47]. Finally, PC1 has contributions from both RORbeta and ER81 layer maps, but in the reverse direction. These genes are enriched in layers 4 and 5, which are generally considered as the input and output layers [61]. We speculate that the maturation of the structures of layers 4 and 5 is coordinated so that the primary sensory areas are specialized for input reception.

Compared with the interpretation of PC1, that of PC2 is more difficult. We could think of possible developmental causes for the conspicuous lateral-to-medial gradient of PC2: it may reflect cortical patterning by a gradient of regulatory genes [58], [62], or a cortical migratory stream [63]. However, the functional significance of such a gradient in the mature neocortex remains unclear. Arimatsu and coworkers report that Nurr1-positive neurons send corticocortical but not corticothalamic projections in the rat cortex [19]. We found that this projection specificity is also conserved in monkeys [23]. These observations suggest that PC2 may represent a special type of cortico-cortical connectivity.

It is quite intriguing that Nurr1 mRNA is expressed by a subtype of neurons distinct from those expressing RORbeta or ER81 mRNAs despite the extensive intermingling (Fig. 3). The negative correlations of Nurr1 with RORbeta or ER81 contribution in PC1 and PC2 (Fig. 7A) raise the possibility that the same organizing principle differentially affects different cell types. We predict that there may be some rules that coordinate the expression of thousands of genes in organizing the neocortical structure. How such coordination occurs is, at present, an open question. The double ISH and cortical box method are useful tools for analyzing such rules and for revealing the principles behind them.

## Materials and Methods

### Animals and tissue preparation

Four adult male Sprague Dawley rats (one for double ISH, three for cortical box method of single ISH) were purchased from Japan SLC, Inc. (Hamamatsu, Japan) and perfused through the heart with 4% paraformaldehyde in 0.1 M phosphate buffer (pH 7.4) under deep anesthesia induced by Nembutal (50 mg/kg body weight, i.p.). All the experiments were conducted in accordance with the Guide for the Care and Use of Laboratory Animals (National Institute of Health (USA) publication number 86–23, 1985) and the guidelines of the Okazaki National Research Institutes in Japan. We made all efforts to minimize the number of animals used and their suffering.

### Probe preparation

The cDNA fragments of mouse or rat RORbeta were obtained by polymerase chain reaction (PCR) using the primers listed in Table 1 and subcloned into the pBlueScriptII vector. Each of the three probes listed in Table 1 exhibited very similar expression patterns for both mouse and rat brains (data not shown), validating the reproducibility of the ISH result. To obtain the data presented in this paper, we used two probes, rRORbeta2 and rRORbeta3, mixed together for hybridization. The probes for the ER81 and Nurr1 gene were previously described [21]. The digoxygenin (DIG)- and fluorescein (FITC)-labeled riboprobes were produced by in vitro transcription using these plasmids as templates. The riboprobes were purified using ProbeQuant 50 spin column (Amersham Biosciences, Little Chalfont, UK).

**Table 1:** Primers used to clone RORbeta gene segments.

Plasmid name	Primer set	template	Target sequence (CDS = +1 to +862)
mRORbeta	GTGTACAGCAGCAGCATTAGCA	Mouse brain	From −136 to +676
	GGTCTCATCATCCAGGTGRTTC	cDNA	
rRORbeta2	AAAGCAAGCACATTGGAGAG	Rat brain	From −840 to −93
	GTCAATGACGTGCCCGTTGG	cDNA	
rRORbeta3	AACAAACAGAAGAGCCCCAC	Rat brain	From −73 to +1029
	GCCAACGGGCACGTCATTGACC	cDNA	

### Single-color ISH

A silicon template was used to cut vertically the brain into half (anterior and posterior parts). Coronal sections from both hemispheres were cut at 40 µm thickness using a freezing microtome. Special care was taken to maintain the orientation of sections. Every seventh section was preserved for ISH using RORbeta, ER81 and Nurr1 gene probes and Nissl staining. The remaining sections were frozen for later use. These sections were equivalent to the entire hemisphere with an approximately 280 µm interval. ISH was carried out as previously described [21], [64]. Briefly, free-floating sections were treated with proteinase K (1 µg/mL) for 30 minutes at 37°C, acetylated, then incubated in a hybridization buffer containing 0.25–0.5 µg/mL digoxigenin-labeled riboprobes at 60°C. The sections were sequentially treated in 2× standard saline citrate (SSC)/50% formamide/0.1% N-lauroylsarcosine for 20 minutes at 60°C, twice; 30 minutes at 37°C in RNase buffer (10 mM Tris-HCl, pH 8.0, 1 mM EDTA, 500 mM NaCl) containing 20 µg/mL RNase A (Sigma-Aldrich, St. Louis, MO); 20 minutes at 37°C in 2× SSC/0.1% N-lauroylsarcosine, twice; 20 minutes at 37°C in 0.2× SSC/0.1% N-lauroylsarcosine, twice. The hybridized probe was detected with an alkaline phosphatase-conjugated anti-DIG antibody using a DIG nucleic acid detection kit (Roche Diagnostics, Indianapolis, IN, USA). There were no apparent signals in control sections examined with the sense probes. ISH causes considerable tissue section shrinkage (80%). However, the scale bars in the figures are not adjusted for such shrinkage.

Double ISH was carried out using DIG- and FITC-labeled riboprobes as previously described [23]. The sections were cut to 15–20 µm thickness. The hybridization and washing were carried out as described above, except that both DIG and FITC probes were used for the hybridization. The fluorescent detection was performed as described using TSA-plus reagent (Perkin Elmer, Wellesley MA, USA) and HNPP fluorescent detection set (Roche diagnostics).

### Image acquisition

The images for the single- and double-color ISH were obtained using a digital color camera DP 70 (Olympus, Tokyo, Japan) attached to a BX-51 microscope (Olympus). For the analysis by cortical box method, digital images (1360×1024 pixels) were captured using the 1.25× objective in the gray scale with 8 bits (10.3 µm/pixel). The background image was subtracted using Adobe Photoshop (Adobe Systems, San Jose, CA) to eliminate the shadowing effect.

### Standardization of regions of cortex

The standardization of the rat cortex was conducted as described previously with a slight modification [31]. To achieve objective and automatic procedures, we restricted the quantification of gene expression to the posterior half of the cortex, which has clear structural landmarks. We used 17 coronal sections in each animal, which were presumed to correspond to the Bregma of −2.1 to −6.3 mm, as determined from the order of serial sections and the shape of the hippocampus [49]. Sections that contain artifacts such as tearing or bubbles in the cortex were excluded from the analysis. The averages of 16.5±0.83 (mean±SD) (ISH for RORbeta), 16.5±0.54 (ISH for ER81) and 15.8±1.17 (ISH for RORbeta) sections processed by ISH for each animal were used for this analysis.

For the standardization of the cortex, the section images were processed as follows. The medial end of the white matter and the valley of the rhinal fissure were chosen as structural landmarks of the mediodorsal (MD) and lateroventral (LV) ends of the cortical sections, respectively. The pial surface and the border between the cortex and the white matter were chosen as the outer (OC) and inner contours (IC), respectively. Then, the image of a large part of the cortex was manually cut out on the basis of these landmarks using Adobe Photoshop (Fig. 4A). The following steps were automatically carried out using a customized software program designed by LabVIEW 7.0 (National Instruments, Austin, TX, USA). The lengths of OC and IC were measured and equally divided into 100 points. Sectors that were defined by every two adjacent points on each contour were extracted and converted to standardized rectangles by linear interpolation. These rectangles from MD to LV were aligned from left to right to form a “standardized cortical section” (100×1000 pixels, depth and width, respectively, Fig. 4A). The standardized cortical sections are distorted toward deeper layers, because the outer contour is always longer than inner contour. We evaluated the transformation rates of standardized sections from original sections. The average rates of transformation across areas were, 90±5% in layer 2/3, 100±5% in layer 4, 110±5% in layer 5 and 120±5% in layer 6, respectively. The transformation is performed so that the local density of staining is preserved. Therefore, distortion by the transformation does not affect the overall patterns of gene expression. Although our method introduces the transformation especially in deeper layers, the boundaries of the primary sensory areas were the same for both layers 4 (RORbeta) and 6 (Nurr1) (Fig. 5), suggesting that deviation in the deeper layer was only limited.

To normalize the staining intensity, the means and standard deviations (SD) of all the pixel values for the ISH images of one dataset were calculated. These pixel values were converted to 0–100 (%), by linearly adjusting them to mean + 1 SD as 0% and mean + 3 SD as 100%. The data for the sections lost or discarded were generated by linearly interpolating the adjacent serial sections. No attempts were made to count the number of positive cells, different from our previous study [31], because we considered that the staining intensity, which reflects the relative mRNA abundance, was most important in evaluating the gene expression patterns in the current study. Seventeen standardized cortical sections were aligned from the posterior to the anterior cortex to construct a “standardized cortical box” (Fig. 4B). To generate the standardized map of a particular layer (1000×340 pixels) (width and Bregma distances, respectively), the specific layer fraction (10–30% for layer 2/3; 30–50% for layer 4; 50–75% for layer 5; 75–100% for layer 6) was extracted from the standardized cortical box (Fig. 4D) and compressed into a two-dimensional map by averaging. Post hoc smoothing (spatial averaging) was achieved using a moving window operator (41×41 pixels). To create average layer maps, the maps from both hemispheres of all the animals were averaged. To create the CV map, the CV (CV = SD/average×100 (%)) was calculated for each pixel. When the average of a pixel was lower than 10%, the corresponding pixel was covered with white in the CV map, because the average near zero diverges the CV value into infinite. Visualizations were carried out using Matlab 7.0 (Mathworks, Natick, MA, USA).

We plan to open the source code of cortical box method on our website. Researchers who are interested in using this method are welcome to ask details of our method before the website opening.

### Cortical layer and area identification

To determine the location of cortical layers and areas in the standardized sections, we also applied essentially the same procedure described above to a series of adjacent Nissl-stained sections (17 sections for each animal). The local density of neurons was expressed as a GLI, which indicates the pixel intensity of each image [36]–[38]. To correct intersection differences in staining intensity, the intensity was normalized for each section by linearly adjusting the mean as 0% and mean +0.5 SD as 100 (%). To reduce the artifact due to staining variance, the normalized pixel values of every three adjacent standardized sections were averaged to make one averaged standardized section. Each standardized map was averaged to determine the average GLI (cytoarchitectonic) distribution for all the animals. Cortical layers were identified on the basis of the local peaks of the GLI layer profile (Fig. 4C). The Nissl-standardized layer 4 map was constructed by extracting layer 4 fraction (30–50%) from the standardized cortical box (Fig. 4D). Because the primary sensory areas have higher cell densities in layer 4 [8], the borders of these areas were delineated by tracing the local highest rate of GLI changes on the layer 4 map. As a result, primary somatosensory (Parietal cortex, area1 (Par1)), auditory (Temporal cortex, area1 (Te1)), and visual (Occipital cortex, area1 (Oc1)) areas were cytoarchitectonically identified as the regions that had the highest cell densities.

### Data analysis of standardized cortical map

Analyses of standardized cortical maps were performed using Matlab and LabVIEW. For linear regression analysis, the correlation coefficient and a p-value for testing the hypothesis of no correlation between two images were calculated. For PCA, we used 5 representative data sets from the standardized layer maps (layer 4 for RORbeta, layers 4–5 for ER81, layers 5–6 for Nurr1) shown in Fig. 6B. The other maps were excluded from this analysis because they had no or very low signals. The standardized layer maps were analyzed as a P x N matrix (row x column), where P is the location number (P = 340,000 pixels (1000×340 pixels)), and N is the number of layer maps (N = 5). Each column of this matrix was normalized using the standard deviation of the data in that column. The correlation matrix (340,000×340,000) was computed using the normalized data set. PCA was used to determine the eigenvalues and eigenvectors of the correlation matrix [65]. The eigenvalue is the sample variance of the projected data points. The components of the eigenvector are the cosines of the angles between the original variable axis and the corresponding principal axis. PCA seeks the order of determinants of a linear combination of the original variables so that the variance of the resulting values is maximum. The components of the eigenvectors provide the coefficients that define the linear combination, while the resulting scores are the projected points. The first two primary components with SDs higher than those of standardized original images (eigenvalues: PC1, γ = 1.87; PC2, γ = 1.50) are shown in Fig. 7A.

### Nomenclature

For cortical regions other than Ect, the nomenclature followed that of Palomero-Gallagher and Zilles [8]. This reference was chosen because their definitions of cortical areas were partially derived from the GLI analysis that was also employed in our study. In some cases (Ect and DZ), the nomenclature was related to those used in the stereotaxic atlas of Paxinos and Watson [49] because not only is this a commonly used tool in neuroscience research for the rat cortex, it is also consistent with our result.
